# Perceptions of pharmacist-furnished nicotine replacement therapy among participants who smoke in California

**DOI:** 10.1016/j.japh.2025.102450

**Published:** 2025-07-06

**Authors:** Sara Schneider, Arturo Durazo, Sarina Rodriguez, Alec M. Chan-Golston, Tanner Wakefield, Deanna M. Halliday, Darrin Tracy, Anna V. Song, Dorie E. Apollonio

**Affiliations:** Postdoctoral Scholar, Nicotine and Cannabis Policy Center, Health Sciences Research Institute, University of California, Merced, Merced, CA; Assistant Professor of Public Health, Director of the Nicotine and Cannabis Policy Center, Nicotine and Cannabis Policy Center, Health Sciences Research Institute, University of California, Merced, Merced, CA; and Department of Public Health, School of Social Sciences, Humanities and Arts, University of California Merced, Merced, CA; Doctoral Student, Department of Public Health, School of Social Sciences, Humanities and Arts, University of California Merced, Merced, CA; Assistant Professor of Public Health, Director of Biostatistics and Data Support Center, Department of Public Health, School of Social Sciences, Humanities and Arts, University of California Merced, Merced, CA; and Biostatistics and Data Support Center, Health Sciences Research Institute, University of California, Merced, Merced, CA; Associate Specialist, UCSF Cardiovascular Research Institute, Center for Tobacco Control Research and Education, University of California, San Francisco, CA; and School of Medicine, University of California, San Francisco, San Francisco, CA; Postdoctoral Scholar, UCSF Cardiovascular Research Institute, Center for Tobacco Control Research and Education, University of California, San Francisco, CA; Lecturer, Pharmacist, Department of Psychological Sciences, School of Social Sciences, Humanities and Arts, University of California, Merced, Merced, CA; and Nicotine and Cannabis Policy Center, Health Sciences Research Institute, University of California, Merced, Merced, CA; Professor of Psychology, Associate Vice Provost for Academic Personnel, Nicotine and Cannabis Policy Center, Health Sciences Research Institute, University of California, Merced, Merced, CA; Research Consultant, Nicotine and Cannabis Policy Center, Health Sciences Research Institute, University of California, Merced, Merced, CA; Professor, School of Pharmacy, University of California, San Francisco, San Francisco, CA; and Visiting Fellow, Global Health Centre, Graduate Institute of International and Development Studies, Geneva, Switzerland

## Abstract

**Background and objective:**

California’s Central Valley has high rates of tobacco product use and low rates of access to primary care providers. In 2016, California sought to increase access to cessation treatment by allowing pharmacists to prescribe nicotine replacement therapy (NRT). We sought to identify the extent to which this prescribing authority has been integrated into practice.

**Methods:**

From December 2023 to May 2024, we surveyed adult California participants (*n* = 271) who smoke about their smoking patterns, perceptions towards NRT, experiences with receiving tobacco cessation resources in pharmacies. Participants were recruited via email and in person. We analyzed participants’ smoking and quitting history, perceptions of NRT, and experiences with tobacco cessation, comparing residents of California’s Central Valley (*n* = 52) to other regions of the state (*n* = 219).

**Results:**

Smoking rates were comparable for respondents in the Central Valley and those residing in other regions of California, although older respondents tended to smoke more heavily. Respondents had few positive perceptions regarding NRT and expressed concerns about perceived side effects and the risk of dependency; past use of NRT was also associated with lower odds of quitting.

**Conclusions:**

Despite the low risks and high efficacy of prescription NRT for tobacco cessation, participants in our sample expressed concerns about its perceived side effects, potential for dependency, and a belief that it was not useful. Our findings suggest that additional efforts are needed to improve education about NRT, such as via pharmacist-provided tobacco cessation services.

## Background

California’s Central Valley has one of the highest rates of tobacco product use in the state, where as of 2023, 14.6%–21.1% of residents had used any tobacco products within the past 30 days,^1,2^ compared to regions like the Bay Area where the rate was 9%.^3^ Combined with one of the lowest rates of access to traditional primary care providers, it is challenging for residents to obtain effective tobacco cessation treatment,^4,5^ and may be particularly important for older adults who tend to smoke more heavily than younger adults.^6^ Access to primary care providers is low in part due to a shortage of physicians and specialists in the region, making it effectively a medical desert.^7,8^ In areas with limited primary care services like the Central Valley, people often obtain care from local pharmacies, which can be visited without an appointment, do not require payment for health advice, and are open beyond traditional business hours.^9–11^ This combination of characteristics has made pharmacies providers of first resort for medically indigent populations facing exclusion from traditional models of care.^12^

To address nicotine addiction nationally, the United States allows for the sale of both over-the-counter (OTC) and prescription nicotine replacement therapy (NRT) products in pharmacies, but past research has found that the use of NRT alone (ie, without counseling) does not improve tobacco abstinence.^13–15^ However, the provision of NRT, combined with counseling by pharmacists, has been found to double or triple tobacco abstinence rates relative to NRT use alone in community practice settings.^16–19^ This combination of medication and counseling has the potential to increase tobacco cessation in regions where access to care from physicians is limited,^16–19^ but a gap exists in our understanding of how people who smoke perceive NRT and their access to it through pharmacies.

In 2016, California joined New Mexico in authorizing pharmacists to prescribe NRT, including products and doses that are not available OTC, a process referred to in California as “furnishing.”^20^ For NRT, this process involves the pharmacist identifying who can benefit from NRT, reviewing their tobacco use, obtaining their history of previous quit attempts, identifying appropriate products, providing recommendations for additional assistance (eg, state helpline), answering questions, notifying the patient’s primary care provider, documenting the NRT product furnished as a prescription, and retaining documentation for at least 3 years.^21^ In 2019, California also began allowing pharmacists to submit insurance claims for reimbursement for prescribing and counseling prescription—but not OTC—NRT as providers without a supervising physician.^12,22^ Medi-Cal is the only program which explicitly reimburses pharmacists, but private insurers may potentially choose to do so.^23^ As of 2024, 20 states authorized pharmacists to prescribe tobacco cessation pharmacotherapy, including NRT.^24^

Despite the efficacy of NRT, racial and ethnic minority populations who smoke are less likely to use NRT, but these populations appear more likely to use these products when given culturally and linguistically appropriate care.^25^ In this regard, pharmacist furnishings can be an important force in promoting smoking cessation. Individuals who seek care from furnishing pharmacists can access higher dose products and additional cessation modalities (e.g., NRT nasal spray, historically), and receive counseling, collectively increasing the likelihood of quitting.^18,21,26,27^ In California, the availability of pharmacist furnishing for different medications (eg, hormonal contraception, naloxone, PrEP, and PEP) varies depending on medication type and region, at between 3% and 42% of pharmacies.^28–31^ As of 2018, the California Department of Health Care Services reported that only 1% of paid claims through Medi-Cal were for NRT products, showing that NRT furnishing was utilized less than other medications,^32^ despite patients showing positive perspectives towards pharmacist-furnishing of other medications like PrEP and PEP.^33^

## Objectives

Our primary objective was to examine the perceptions of California residents who smoke towards NRT. Given the broad advertising of NRT as a cessation aid, we anticipated that respondents would have positive perceptions of NRT as an evidence-based quit method. As a secondary outcome, we also sought to identify specific regional needs in the predominantly rural Central Valley.^34^ We anticipated that despite the reliance on pharmacists as primary care providers in the Central Valley,^35^ most residents would not report experience with accessing tobacco cessation resources through pharmacists, despite pharmacists’ ability to prescribe NRT and counsel patients. Our final objective was to analyze if there were differences in smoking patterns based on participants’ region. Here, we anticipated older respondents would smoke more heavily than their younger counterparts, and smoking rates would be higher in the Central Valley. Combined, these objectives were intended to identify factors that need to be promoted or addressed to increase the use of tobacco cessation services in the Central Valley, in part by contrasting them to the attitudes and experiences of Californians residing in other parts of the state. We surveyed participants to assess these outcomes.

## Methods

From December 2023 to May 2024, we deployed a survey to a community-based participant pool of research volunteers. All participants provided consent, and study materials were reviewed and approved by the Institutional Review Board at the University of California San Francisco (IRB number 21–35317). Survey respondents who met study inclusion criteria received a $10 participant incentive in the form of an Amazon e-gift card.

### Data collection

Consistent with other sources,^36^ we defined the Central Valley as 11 counties in the San Joaquin Valley and Sierra Nevada Foothills (Fresno, Kern, Kings, Madera, Merced, San Joaquin, Stanislaus, Tulare; and Calaveras, Mariposa, and Tuolumne). We collected all data using the Qualtrics survey platform (Provo, UT); the survey could be completed from a mobile phone, tablet, or computer. Our data was not preregistered due to the observational, rather than clinical, nature of the study. Two senior authors [initials redacted], each with decades of experience in survey piloting, fielding, and analysis, developed the present survey using questions drawn from validated instruments (see Supplement A). The survey also underwent an external review prior to it being deployed. Given that the instruments had been previously validated, no pilot testing was required.

For our first round of data collection, we recruited potential participants using an existing list of participants in community-based studies conducted by the Nicotine and Cannabis Policy Center (NCPC) at the University of California Merced (UC Merced) who had agreed to be contacted about future research. We recruited participants by email; responses were included only if the email address used matched the existing list. For our second round of data collection, NCPC Tobacco Endgame interns, as well as interns from the California State University, Stanislaus’ Smoke and Vape Free Scholars program, physically recruited potential participants. Interns distributed business cards containing brief information about the study, a QR code link to the survey, and a unique access code to self-reported people who smoke outside local businesses (eg, grocery stores, shopping centers), as well as to people they knew personally who smoked, including people from their hometowns. A third and final round of data collection recruited Central Valley residents participating in focus groups on tobacco cessation; those who expressed interest in completing the survey were provided with an email containing a unique access code.

### Measures

Survey questions included closed-ended questions that asked participants about their experiences with smoking, perceptions towards using NRT products, and experiences with seeking tobacco cessation resources from pharmacists in California (see Supplement A). Initial screening questions assessed whether participants were eligible (eg, a person who currently smokes), followed by a series of cigarette use questions derived from the Tobacco Use Supplement of the Current Population Survey.^37^ We asked participants about perceptions of the benefits and drawbacks of NRT using questions from the Attitudes Toward Nicotine Replacement Therapy Scale,^38^ followed by questions derived from the CDC’s Ask, Advise, Refer guidelines for pharmacies/pharmacists,^39,40^ modified to be relevant to consumers, in order to gauge their opinions of and experiences with pharmacy-based cessation services. Participants were asked about their smoking knowledge and attitudes using questions from the Smoking Knowledge, Attitudes, and Practices questionnaire.^41^ Finally, participants were asked demographic questions drawn from the Behavioral Risk Factor Surveillance System.^42^ Minor phrasing changes and modifications were made to questions as needed for online use (eg, rephrased open-ended questions for interviews into closed-ended formats with scaled responses).

Outcome measures included cigarette packs per day, broken down into 4 categories (a few cigarettes, less than half a pack, half a pack to a pack, over a pack) to account for the skewness of the majority of participants (76%) consuming a few or less than half a pack of cigarettes a day. Measures for participants’ experiences with tobacco cessation at pharmacies included whether participants had received advice at a pharmacy on how to quit smoking, or if someone at a pharmacy had provided a referral to a smoking cessation clinic or specialist, a referral to a free quit line, or educational materials about smoking. Measures for participants’ perceptions of NRT included benefits (e.g., NRT helps people to quit smoking) and drawbacks (eg, concerns about side effects) participants identified using NRT. Participants were defined as having made a quit attempt if they had quit smoking voluntarily for at least 24 h within the past year.

### Data analysis

All survey responses were de-identified and screened for validity. The first round of data collection, which relied on past participants in UC Merced research studies, ultimately included respondents residing within and outside the Central Valley. Surveys from California residents who resided outside the Central Valley were used as a comparison with Central Valley residents; we classified respondents’ residences by their latitude/longitude, which was automatically collected by the survey software. The survey was expected to take approximately 20 min to complete; responses were therefore omitted if they were completed in less than 120 s. Responses were also omitted if the same respondent resubmitted a survey with different responses or a respondent did not reside in California. Surveys from the second and third rounds of data collection, recruited by in-person contact, were excluded if their recorded latitude/longitude fell outside of the Central Valley, if responses were completed in less than 120 s, or if responses were resubmitted with different responses and without unique access codes (eg, 1 participant identified by identical latitude/longitude submitted 16 surveys with differing responses).

In addition to reporting descriptive data, we assessed the extent to which participants were aware of pharmacist NRT prescribing, including whether they had ever obtained an NRT prescription through a pharmacist, and their perceptions of NRT. Summary statistics of all variables were calculated and differences in demographics by region were evaluated using chi-square tests. We also investigated differences in smoking patterns between Central Valley residents relative to other California residents by using demographic variables ANOVA models and chi-square tests. All analyses were completed using Stata version 18.0.

## Results

### Description of the sample

After exclusions, our sample contained 52 respondents residing in the Central Valley, and 219 respondents residing in other regions in California, for a total of 271 participants. We received from 9 of the 11 Central Valley counties; the missing Sierra Foothills counties (Mariposa [2023 population = 16,919^43^] and Tuolumne [2023 population = 54,204^44^]) both had fewer residents compared to larger counties in the Central Valley (e.g., Fresno’s 2023 population was 1.017 million^45^; see Supplement B for county-level data). Within our sample, Central Valley respondents were more likely to report being Latinx (31%) compared to respondents in other regions of California (4%; see Table 1). Respondents in other regions of California were more likely to report being White, who may or may not have identified as Hispanic (76%), compared to those in the Central Valley (71%). Reported packs per day were comparable for respondents in the Central Valley and those residing in other regions of California.

### Perceptions of NRT

A minority of all respondents reported beliefs that NRT helped people quit smoking (Central Valley [44.2%]; other regions [35.2%]), helped reduce irritation (Central Valley [36.5%]; other regions [35.6%]), anxiety (Central Valley [30.8%]; other regions [38.4%]), and depression during quit attempts (Central Valley [42.3%]; other California regions [32.4%]), or helped people resist the urge to smoke (Central Valley [19.2%]; other California regions [20.1%]; see Table 2). Significantly more Central Valley residents (51.9%) reported that NRT helped people cope with cravings for cigarettes than those residing in other regions of California (35.2%; *P* = 0.025). With respect to perceived drawbacks of NRT, a minority of Central Valley respondents (42.3%) expressed concerns about side effects compared to a majority of residents from other regions of California (56.2%). A majority of all respondents perceived that NRT use carried a risk of dependency (Central Valley [53.9%]; other regions [64.4%]), and indicated that that they did not need NRT to quit smoking (Central Valley [28.9%]; other regions [37.4%]). Respondents from other California regions reported significantly more concern (42.3%) about using NRT than respondents from the Central Valley (62.6%; *P* = 0.008).

### Experiences with tobacco cessation at pharmacies

**Figure 1.**
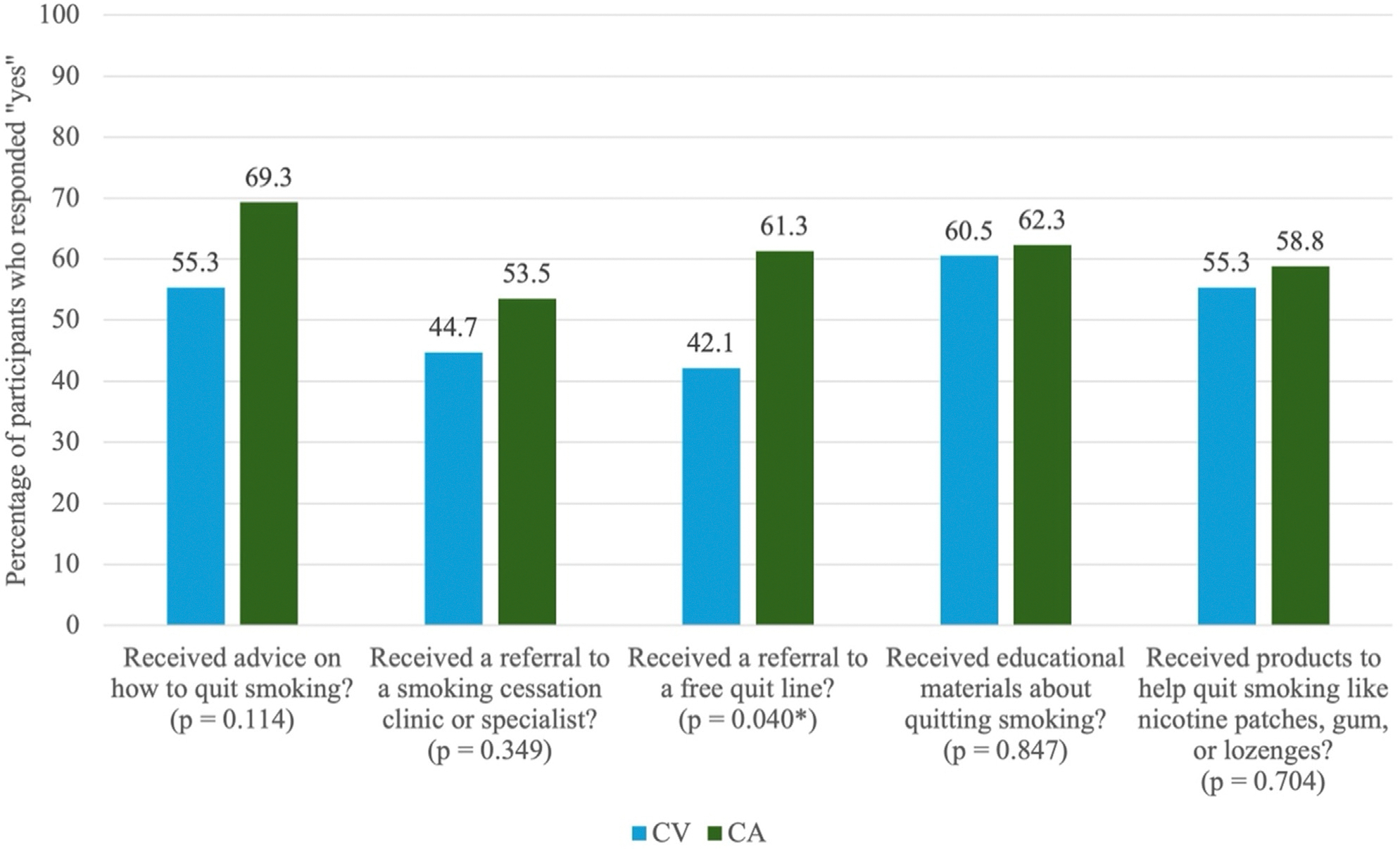
Pharmacy-related experiences with quitting smoking ***P < 0.001, **P < 0.01, *P < 0.05. CV, participants who reside in the Central Valley; CA, participants who reside in other regions of California.

Few regional differences were reported between respondents residing in the Central Valley and those in other regions of California when assessing experiences with tobacco cessation at pharmacies (see Figure 1). Slightly more respondents from both groups reported having received advice on how to quit smoking (Central Valley [55.3%]; other regions [69.3%]), educational materials about quitting (Central Valley [60.5%]; other regions [62.3%]), or products to help quit from a pharmacy (Central Valley [55.3%]; other regions [58.8%]). Fewer Central Valley respondents (44.7%) reported having ever received a referral to a smoking cessation clinic or specialist at a pharmacy than respondents in other California regions (53.5%), but the difference was not significant. Respondents from outside the Central Valley (61.3%) were significantly more likely to report having received a referral to a free quit line than those from within the Central Valley (42.1%, *P* = 0.040).

### Smoking patterns by age

Comparisons of packs smoked per day by age were assessed using an F-test from an ANOVA model. Significant differences in age were observed (see Table 3), with a trend suggesting that participants who smoked over a pack per day were significantly older compared to those who smoked less than a pack per day. However, as a sensitivity analysis, we conducted pairwise comparison of all age group means in the full dataset. It suggested that participants smoking “over a pack” were the only significantly different group in terms of average age (they were older on average than other participants who smoke). This pattern was consistent for both the Central Valley residents and those residing in other regions of California.

## Discussion

We surveyed participants who smoke and reside in both the Central Valley and in other regions in California about their experiences with smoking, perceptions towards using NRT products, and their experiences with receiving tobacco cessation resources in pharmacies. Similarly to prior studies,^6^ respondents from both within and outside the Central Valley who smoked more heavily were older and had lower odds of quitting than those who smoked less heavily, although age alone was not associated with the likelihood of quitting. Respondents from both regions had similar experiences overall with tobacco cessation at pharmacies, but more respondents residing outside the Central Valley had received a referral to a free quit line than those residing in the Central Valley. Thus, although Central Valley residents smoke at similar, if not higher rates,^1–3^ they are less likely to be referred to cessation support. One of the goals of allowing NRT furnishing by pharmacists was to provide increased access by giving community pharmacists the ability to counsel on tobacco cessation, in recognition of their accessibility and expertise. However, to date, this expectation does not appear to have been realized in the Central Valley region. Additional cessation support in the region may be effective in reducing smoking prevalence.

With the exception of Central Valley residents reporting that NRT helped people cope with cigarette cravings, only a minority of respondents, regardless of residence, reported positive perceptions of NRT (e.g., NRT does not help people quit smoking). Instead, respondents expressed concerns about NRT’s side effects and risk of dependency, and reported that they did not need NRT to quit. These findings stand in contrast to studies which have analyzed perceptions towards furnishing of other medications like PrEP and PEP,^30^ suggesting that residents may be open to pharmacist-led services, but have reservations about NRT specifically.

Despite the known risks of smoking, respondents expressed concerns about the perceived risks of using NRT (eg, concern about NRT’s side effects). These perceptions are inconsistent with the extensive research demonstrating that NRT, when combined with counseling, aids in cessation, has minimal risks (eg, skin rash, upset stomach), and is safe to use.^46^ These findings may be, at least in part, a driving factor behind why so few pharmacies were previously found to furnish NRT,^32^ and suggest that more outreach and education would be needed to increase uptake of NRT and improve cessation outcomes.

California and other states have identified pharmacists and pharmacies as an ideal point of contact, and consistent with this expectation, some respondents reported receiving advice and educational materials about cessation from pharmacists. However, these services appear not to have extended to education about NRT and its potential to improve cessation outcomes. Misperceptions about NRT, as well as reports of past unsuccessful cessation use, may reflect known problems with attempting to quit using OTC NRT. Past research suggests that without counseling, people who use NRT may be unsuccessful due to inappropriate dosages or incorrect use (eg, not using for the recommended time period, not using the “chew and park” method).^47^

Our study has limitations. Part of our study relied on online survey methodology, which has well-documented risks and benefits.^48^ Moreover, for the first round of data collection, the participant list contained more participants than anticipated who appeared to have moved out of the Central Valley or out of California, highlighting the need to maintain and update lists of past study participants willing to participate in future studies by maintaining regular contact. We addressed these limitations, in part, by expanding to in-person recruitment in the second and third rounds of data collection. Although our third round of data collection excluded smokers who are not interested in quitting, approximately two-thirds of smokers have a desire to quit smoking,^49^ so a limited amount of smokers would have been excluded due to this criteria. The survey was only provided in English due to translation constraints, although other languages, including Spanish, are commonly spoken in the region. Our reliance on a written survey, which was chosen for consistency, may have prevented some persons from participating, especially given the Central Valley’s high rates of illiteracy (eg, for adults in Tulare County, functional illiteracy in English has been reported to be as high as 41%^50,51^). Future studies conducted verbally or in other languages may identify different communities that have different perceptions or experiences with NRT and pharmacist prescribing and education.

## Conclusion

Despite the low risks of NRT and its efficacy for cessation when combined with counseling, participants who smoked were concerned about potential side effects and dependency, did not perceive that they needed NRT to quit, and reported that past NRT use was associated with lower odds of quitting. Multiple states, including California, have sought to increase access to tobacco cessation by allowing pharmacists to prescribe medications, but few participants who smoke reported experience with these services, suggesting that they may have relied on OTC NRT instead of receiving appropriate doses and information about how to use products from a healthcare professional. Additional public outreach and educational initiatives at pharmacies may be needed to increase furnishing and tobacco cessation. For example, pharmacies that prescribe hormonal contraception frequently advertise this service on site; similarly, multiple pharmacies advertise access to vaccination services both inside and outside stores. Future research should consider the potential for expanded advertising and education at pharmacies to increase public knowledge about the role of NRT in tobacco cessation and increase tobacco cessation in high-need regions.

## Supplementary Material

Supplement 1

Supplement 2

Supplementary data

Supplementary data related to this article can be found at https://doi.org/10.1016/j.japh.2025.102450.

## Figures and Tables

**Table 1:** Demographic data for participants who reside in the Central Valley and in other regions of California

Variable	Frequency (%)		*P* value
	CV *n* = 52	CA *n* = 219	

Age	31 (*SD* = 14)	33 (*SD* = 12)	0.3042
Sex			0.116
Male	24 (46.4)	112 (51.1)	
Female	25 (48.1)	107 (48.9)	
Latinx			<0.001***
Yes	16 (30.8)	9 (4.1)	
No	36 (60.2)	209 (95.4)	
Specific Latinx (Only if Latinx = yes)			0.444
MX/Chicano	15 (93.8)	9 (100)	
Race/ethnicity			<0.001***
White/Caucasian	37 (74.00)	172 (78.8)	
Asian	0	24 (11.0)	
Black/African American	1 (2.00)	11 (5.0)	
Other	12 (24.00)	9 (4.1)	
Specific Asian (Only if Asian = yes)			<0.001***
Asian Indian	0	18 (52.9)	
Chinese	1 (10.00)	10 (29.4)	
Other	6 (60.00)	0	
Born in U.S.			
Yes	51 (98.1)	216 (98.6)	0.270
Preferred language			
English	51 (98.1)	217 (99.1)	0.532
English fluency			0.399
Very well	49 (94.2)	203 (92.7)	
Well	2 (4.0)	15 (6.8)	
Number of people in household			0.170
1	12 (23.1)	30 (13.7)	
2	10 (19.2)	38 (17.4)	
3	11 (21.4)	73 (33.3)	
4	9 (17.3)	49 (22.4)	
5	6 (11.5)	21 (9.6)	
6	3 (5.8)	7 (3.2)	
Income			<0.001***
$10,000–$14,999	7 (13.5)	32 (14.6)	
$15,000–$19,999	2 (3.9)	31 (14.2)	
$20,000–$34,999	8 (15.4)	34 (15.5)	
$35,000–$49,999	10 (19.2)	40 (18.3)	
$50,000–$74,999	10 (19.2)	45 (20.6)	
$75,000–$99,999	3 (5.8)	30 (13.7)	
$100K +	6 (11.5)	6 (2.7)	
Packs smoked			0.996
A few cigarettes	12 (23.1)	50 (22.8)	
Less than 1/2 pack	28 (53.9)	115 (52.5)	
1/2 pack to a pack	10 (19.2)	45 (20.6)	
More than 1 pack	2 (3.9)	9 (4.1)	

Abbreviations used: CV, participants who reside in the Central Valley; CA, participants who reside in other regions of California.

Note:

*** *P* < 0.001,

** *P* < 0.01,

* *P* < 0.05.

Significant differences in each covariate by region were assessed using chi-square tests. Variables with fewer than 3 total responses (across CV and CA) are excluded from the table. Therefore, data does not sum to 100% due to missing data. See Supplement A for all variables, and Supplement B for county-level data.

**Table 2:** Perceptions of nicotine replacement therapy

Variable name	Frequency (%)		*P* value
	CV *n* = 52	CA *n* = 219	

Perceptions of the benefits of NRT (Select all that apply)			
NRT helps people to quit smoking			0.223
Yes	23 (44.2)	77 (35.2)	
NRT helps people feel less irritable when they quit smoking			0.901
Yes	19 (36.5)	78 (35.6)	
NRT helps people to feel less anxious when they quit smoking			0.308
Yes	16 (30.8)	84 (38.4)	
NRT helps people to feel less depressed when they quit smoking			0.177
Yes	22 (42.3)	71 (32.4)	
NRT helps people to resist the need to smoke in situations where smoking is NOT possible			0.889
Yes	10 (19.2)	44 (20.1)	
NRT help people to cope with the craving for cigarettes			0.025*
Yes	27 (51.9)	77 (35.2)	
Perceptions of the cons of NRT (Select all that apply)			
I am concerned about the side effects of NRT			0.072
Yes	22 (42.3)	123 (56.2)	
I am wary of NRT			0.008**
Yes	22 (42.3)	137 (62.6)	
There is risk of becoming dependent on NRT			0.159
Yes	28 (53.9)	141 (64.4)	
I do NOT need NRT in order to quit smoking			0.245
Yes	15 (28.9)	82 (37.4)	

Abbreviations used: CV, participants who reside in the Central Valley; CA, participants who reside in other regions of California.

Note:

*** *P* < 0.001,

** *P* < 0.01,

* *P* < 0.05.

Significant differences in each covariate by region were assess using chi-square tests. Only “yes” responses are reported.

**Table 3:** Average age by packs smoked per day

Cigarettes smoked per day	Number of participants (%)		Mean age (standard deviation)			*P* value
	CV *n* = 52	CA *n* = 219	All respondents *n* = 271	CV *n* = 52	CA *n* = 219	

A few cigarettes	12 (23.1)	50 (22.8)	31.94 (7.88)	28.42 (7.35)	32.78 (7.84)	
Less than half a pack	28 (53.8)	115 (52.5)	32.82 (7.33)	30.96 (7.66)	33.25 (7.21)	Overall: 0.002**
Half a pack to a pack	10 (19.2)	45 (20.5)	32.75 (8.80)	35.40 (7.82)	32.10 (8.99)	CA: 0.043*
Over a pack	2 (3.8)	7 (3.2)	41.73 (6.59)	49.00 (5.66)	40.11 (5.84)	CV: 0.004**

Abbreviations used: CV, participants who reside in the Central Valley; CA, Participants who reside in other regions of California.

Note:

*** *P* < 0.001,

** *P* < 0.01,

* *P* < 0.05.

Significant differences in age for each group (overall, CV, and CA) were assessed using an overall F-test from an ANOVA model. Note: Two responses were missing from the CA group; percentages are calculated based on nonmissing responses.

## Key Points

### Background:

- Pharmacists are providers of first resort for populations facing exclusion from traditional models of care.
- California pharmacists are able to prescribe or “furnish” nicotine replacement therapy (NRT), benefitting residents of rural regions where smoking rates are high.

### Findings:

- We assessed perceptions of pharmacist-furnished NRT in California, including the Central Valley, where there are high rates of tobacco use and a health professional shortage.
- Our study highlights that California residents who smoke have misperceptions about the benefits and risks of NRT.
