# Supplementary material for: Perceptions of pharmacist-furnished nicotine replacement therapy among participants who smoke in California
Source: J Am Pharm Assoc (2003). Author manuscript; Available in PMC 2026 Mar 20. (PMC13004478; doi:10.1016/j.japh.2025.102450)
Supplement: Supplement 1 [file NIHMS2146351-supplement-Supplement_1.docx]

## **SUPPLEMENT A: SURVEY INTRODUCTION AND QUESTIONS**

## **Introduction**

## This survey aims to understand your current knowledge, opinions, and experiences with Nicotine Replacement Therapy (or medications used to help people quit smoking) through local pharmacies in the Central Valley. The survey should take about 20 minutes to complete, and you will receive a $10 Amazon or Target gift card in appreciation of your participation. To be eligible for this survey, you must be 18 years or older and be a current cigarette smoker. At the end of the survey, you will be redirected to a separate form to report your name and email in order to receive the gift card. Because this information is collected separately, your name and email will not be linked to the responses you make here. We sincerely value your honest responses, and appreciate you taking this time to complete this survey.

**Screening questions**

1. Before we begin, please confirm that you are not a robot. [CAPTCHA response]

2. Are you 18 years or older? (yes/no)

3. Are you a current cigarette smoker? (yes/no)

**Cigarette use (Tobacco Use Supplement of the Current Population Survey)**^34^

Congratulations! You are eligible for this study. We'll begin by asking some questions about your cigarette use.

4. First, how old were you when you first started smoking cigarettes fairly regularly? [Text]

5. Have you smoked at least 100 cigarettes in your lifetime? (yes/no)

6. During the last 30 days, on about how many days did you smoke a cigarette? [Text]

7. How many cigarettes did you smoke in the last 24 hours? [Text]

8. On average, how many cigarettes a day do you smoke? [Text]

9. How soon after waking up do you have your first cigarette?

a. Within 5 minutes

b. 6-30 minutes

c. 31-60 minutes

d. After 60 minutes

10. For how many years have you smoked? [Text]

11. If you quit smoking, when did you quit?

a. Within the last month

b. 1-3 months ago

c. 3-6 months ago

d. 6-12 months ago

e. More than 12 months ago

f. Not applicable

12. In the past year, did you quit smoking voluntarily for at least 24 hours? (yes/no)

12A. If yes, how many times?

13. Are you seriously thinking of quitting smoking?

a. Yes, within the next 30 days

b. Yes, within the next 6 months, but not in the next 30 days

c. No, I am not thinking of quitting within the next 6 months

14. Have you ever used any of the following products to help you try to quit smoking? Please select all that apply.

☐ Nicotine patch

☐ Nicotine gum

☐ Bupropion (Zyban)

☐ Varenicline (Chantix)

☐ Nicotine lozenges, nasal spray, or inhaler

☐ E-cigarettes or vape pens

☐ Something else [text]

15. People reduce or quit smoking for a variety of reasons. How much did/do the following factors affect your smoking/quitting? [slider response]
(1 = not at all; 3 = a moderate amount; 5 = a great deal)

a. Cost of a pack of cigarettes

b. Health concerns

c. Family (pressure, encouragement, being a good role model)

d. Warning labels on cigarette packages

16. During your entire life, have you ever used e-cigarettes, even one or two puffs? (yes/no)

16A (if 16 = yes). During your entire life, about how frequently have you used e-cigarettes?

a. Only once

b. A few times in my life

c. Sometimes

4. Most of the time or always

16B (if 16 = yes). Have you used a cigarette in the past 30 days? (yes/no)

16C (if 16 = yes). In the last 30 days, on about how many days did you use e-cigarettes?

16D (if 16 = yes). Please select the reason(s) why you have used an e-cigarette in the past 30 days:

☐ To reduce/quit cigarettes

☐ At times when I can’t smoke

☐ To reduce health risks

☐ Enjoy the flavor/taste

☐ For a different reason [text]

**Attitudes toward nicotine replacement therapy scale (ANRT-12)**^35^

17. We next want to know your thoughts about Nicotine Replacement Therapy, also known as NRT.

What do you think are the benefits of Nicotine Replacement Therapy (NRT)? Please select all that apply.

☐ NRT helps people feel less irritable when they quit smoking

☐ NRT helps people to feel less depressed when they quit smoking

☐ NRT help people to cope with the craving for cigarettes

☐ NRT helps people to quit smoking

☐ NRT helps people to feel less anxious when they quit smoking

☐ Knowing that NRT exists encourages people to try to quit smoking

☐ NRT helps people to resist the need to smoke in situations where smoking is NOT possible

☐ Other [text]

18. What do you think are the drawbacks of Nicotine Replacement Therapy (NRT)? Please select all that apply.

☐ I am concerned about the side effects of NRT

☐ I am wary of NRT

☐ There is risk of becoming dependent on NRT

☐ I do NOT need NRT in order to quit smoking

☐ Other [text]

**Pharmacy-Based Cessation Services**^36,37^

19. How strongly do you agree or disagree with the following statements?

|  | Strongly agree | Somewhat agree | Neutral | Somewhat disagree | Strongly disagree |
| --- | --- | --- | --- | --- | --- |
| Smoking is a personal decision which does not concern the pharmacist | ◯ | ◯ | ◯ | ◯ | ◯ |
| I am aware of community resources to help people quit smoking | ◯ | ◯ | ◯ | ◯ | ◯ |

20. At your pharmacy, did you receive any of the following services or products? Please select all that apply.

|  | Yes | No |
| --- | --- | --- |
| Advice on how to quit smoking | ☐ | ☐ |
| A referral to a smoking cessation clinic specialist | ☐ | ☐ |
| A referral to a free quit line | ☐ | ☐ |
| Educational materials about quitting smoking such as pamphlets | ☐ | ☐ |
| Products to help you quit smoking such as nicotine patch, nicotine gum, nicotine lozenges/inhalers | ☐ | ☐ |

**S-KAS Knowledge and Attitudes**^38^

21. How strongly do you agree or disagree with the following statements?
(1 = Strongly Disagree; 3 = Neutral; 5 = Strongly Agree) [slider response]

a. I have the required skills to quit smoking

b. I am concerned about smoking

c. Counseling by a pharmacist would help me quit smoking

22. In the past month, how frequently did your pharmacist:
(1 = Never; 2 = Occasionally; 3 = Often; 4 = Very Often) [slider response]

a. Arrange for a follow-up appointment to discuss quitting smoking

b. Encourage you to quit smoking completely

c. Encourage you to use products to help you quit smoking (such as nicotine patch, gum, bupropion (Zyban), varenicline (Chantix))

d. Encourage you to reduce smoking to five or fewer cigarettes per day if you have stated you could not quit

**Demographics**

23. Finally, we would like to ask you about some demographic information.

First, what is your age?

24. What is your current gender identity (check one)?

a. Female

b. Male

c. Female-to-Male (FTM)/Transgender Male/Trans Man

d. Male-to-Female (MTF)/Transgender Female/Trans Woman

e. Genderqueer, neither exclusively male nor female

f. Additional gender category, please specify [text]

g. Decline to answer

25. What sex were you assigned at birth on your original birth certificate (check one)?

a. Male

b. Female

c. Decline to answer

26. Are you of Hispanic or Latino origin?

a. Yes, I am of Hispanic or Latino origin

b. No, I am **not** of Hispanic or Latino origin

26A (if 26 = yes). I am:

a. Central American (please specify: Guatemalan, Nicaraguan, Panamanian, Salvadoran, or Other?) [text]

b. Mexican, Mexican American, Chicano

c. South American (please specify) [text]

d. Another Hispanic, Latino, or Spanish origin (please specify: Cuban, Puerto Rican, or Other?) [text]

27. What is your race/ethnicity? Please select all that apply.

☐ American Indian or Alaska Native (please specify): [text]

☐ Asian

☐ Black or African American

☐ Native Hawaiian or other Pacific Islander

☐ White/Caucasian

☐ Other (please specify)

27A (if Asian is selected). Please specify your Asian identity.

a. Asian Indian

b. Chinese

c. Hmong

d. Filipino

e. Laotian

f. Punjabi

g. Vietnamese

h. Other (please specify) [text]

28. Were you born in the United States? (yes/no)

29. What is your preferred language?

a. English

b. Hmong

c. Laotian

d. Spanish

e. Punjabi

f. Tagalog

g. Other (please specify) [text]

30. How well do you speak English?

a. Very well

b. Well

c. Not well

d. Not at all

31. Including yourself, how many people live in your household? Please enter a number. [text]

32. Thinking about members of your family living in this household, what is your combined annual income meaning the total pre-tax income from all sources earned in the past year?

a. Less than $10,000

b. $10,000 to under $15,000

c. $15,000 to under $20,000

d. $20,000 to under $35,000

e. $35,000 to under $50,000

f. $50,000 to under $75,000

g. $75,000 to under $100,000

h. $100,000 to under $200,000

i. $200,000 or more

j. Don't know

k. Decline to answer
