# Supplementary material for: Perceptions of pharmacist-furnished nicotine replacement therapy among participants who smoke in California
Source: J Am Pharm Assoc (2003). Author manuscript; Available in PMC 2026 Mar 20. (PMC13004478; doi:10.1016/j.japh.2025.102450)
Supplement: Supplement 2 [file NIHMS2146351-supplement-Supplement_2.docx]

## **SUPPLEMENT B: CONTINUED DEMOGRAPHIC DATA: COUNTY**

**Table 1.A. County data for participants who reside in the Central Valley and in other regions of California**

| **County** | **Frequency (*%*)** |
| --- | --- |
| Alameda  Alpine  Amador  Calaveras  Colusa  Contra Costa  El Dorado  Fresno  Humboldt  Imperial  Kern  Kings  Los Angeles  Madera  Marin  Mendocino  Merced  Mono  Nevada  Orange  Placer  Riverside  Sacramento  San Bernardino  San Diego  San Francisco  San Joaquin  San Luis Obispo  San Mateo  Santa Barbara  Santa Clara  Santa Cruz  Stanislaus  Solano  Sonoma  Sutter  Tehama  Tulare  Ventura  Yolo  Yuba | 9 (3.3)  1 (0.4)  1 (0.4)  1 (0.4)  1 (0.4)  10 (3.7)  2 (0.7)  9 (3.3)  1 (0.4)  2 (0.7)  7 (2.6)  2 (0.7)  58 (21.4)  1 (0.4)  2 (0.7)  1 (0.4)  10 (3.7)  2 (0.8)  1 (0.4)  17 (6.3)  4 (1.5)  15 (5.5)  13 (4.8)  5 (1.9)  27 (10.0)  2 (0.7)  7 (2.6)  4 (1.5)  3 (1.1)  4 (1.5)  10 (3.7)  2 (0.7)  14 (5.2)  3 (1.1)  4 (1.5)  1 (0.4)  1 (0.4)  1 (0.4)  10 (3.7)  2 (0.7)  1 (0.4) |
